# Josephine Snetsinger

**Published:** 1905-05

**Authors:** 


JOSEPHINE SNETSINGER, a graduate nurse, recently died at the Buffalo homeopathic hospital. The Buffalo Nurses' Association, of which she was a member, at a recent meeting adopted the following memorial:

WHEREAS, God in His infinite wisdom has taken from us to Himself, our beloved friend and associate, Josephine Snetsinger; and

WHEREAS, The Buffalo Nurses' Association has lost an honored and esteemed member, who ever gave unselfish and devoted service to advancing its interests and to promoting and maintaining a high ideal of nursing and of womanhood; and

WHEREAS, Her memory will always be an inspiration to her friends for loyal, painstaking and conscientious fulfillment of duty. Therefore, be it

*Resolved,* That the Buffalo Nurses' Association hold the memory of Josephine Snetsinger with sentiments of gratitude and affection; and be it further

*Resolved,* That a copy of these resolutions be spread upon the records of this association and a copy sent to the family and friends of our beloved associate; and also to the BUFFALO MEDICAL JOURNAL.

Committee: SYLVEEN V. NYE, ADELAIDE MARSDEN, HARRIET D. STORCK, LOUISE GREENWOOD, BEATA BOWIE.

